# Beacons and BIM Models for Indoor Guidance and Location

**DOI:** 10.3390/s18124374

**Published:** 2018-12-11

**Authors:** Joao C. Ferreira, Ricardo Resende, Stuart Martinho

**Affiliations:** ISTAR-IUL, Instituto Universitário de Lisboa (ISCTE-IUL), Lisboa 1649-026, Portugal; jose.resende@iscte-iul.pt (R.R.); Stuart_Martinho@iscte-iul.pt (S.M.)

**Keywords:** indoor location, mobile app, building information models, BLE, Beacon, Path Finding, A*

## Abstract

This research work uses a simplified approach to combine location information from a beacon’s propagation signal interaction with a mobile device sensor (accelerometer and gyroscope) with local building information to give real-time location and guidance to a user inside a building. This is an interactive process with visualisation information that can help user’s orientation inside unknown buildings and the data stored from different users can provide useful information about users’ movements inside a public building. Beacons installed on the building at specific pre-defined positions emit signals that give a geographic position with an associated imprecision, related with Bluetooth’s range. This uncertainty is handled by building layout and users’ movement in a developed system that maps users’ position, gives guidance, and stores user movements. This system is based on an App (Find Me!) for Android OS (Operating System) which captures the Bluetooth Low Energy (BLE) signal coming from the beacon(s) and shows, through a map, the location of the user’s smartphone and guide him to the desired destination. Also, the beacons can deliver relevant context information. The application was tested by a panel of new and habitual campus users against traditional wayfinding alternatives yielding navigation times about 30% smaller, respectively.

## 1. Introduction

Buildings such as hospitals, schools, shopping centers, or transportation interfaces are increasingly large and complex and therefore hard to navigate efficiently, both for occasional and frequent users. Even when spatial clarity is built into the design, adaptations introduce entropy and compromise the initial intention. Since environmental cues such as sunlight are missing, signage-based orientation, maps, and human-assistance are the traditional methods of orientation. Users usually choose to wander and explore on their own, then ask for directions and sometimes get lost again. It is of the interest of building owners to help users. GPS and inertia based mobile apps are today ubiquitous in outdoor navigation, but GPS is not available indoor. Indoor navigation based on smartphones has been attempted and provides a number of challenges [1]. This life-problem can be easily solved by an Indoor Location System (ILS). An Indoor Positioning System usually implemented via Bluetooth, infrared, magnetic field and/or WIFI, serves the purpose of finding the position of an electronic device-phone, tablet, watch-and, when the smart device connects with the ILS, the system can store that data and use it to inform on the building utilization. A second problem is that indoor maps with relevant, usable information that can be integrated easily in digital apps are usually not available.

This study employs two established technologies to develop a cost-effective solution. A mobile application that resorts to Bluetooth beacons for indoor user localization and Building Information Models (BIM) [2] for physical context. Bluetooth beacons broadcast a Bluetooth Low Energy (BLE) signal in a limited and configurable range. This signal can be interpreted by the mobile device as the location of the user in the building, without the need of internet connection. BIM models, the evolution of traditional Computer-Aided Design (CAD) drawings, associate both three-dimensional geometric and a database of all kinds of information: materials; spaces, or equipment and thus provide necessary context both for the location and navigation. Since most new construction today is based on BIM methodology, the effort for the development of a navigation application using the developed methodology is minimal.

A mobile application—Find Me! App—that integrates Bluetooth beacons and BIM models to show the user his location and the path to the destination was developed for Android smartphones. The most important parts of the Find Me! App are the user’s current location (from the nearest beacon) and the Building Information Model, which holds room, beacon, stair, and elevator locations and navigation maps and delivers the location of the destination. Beacons are configured with identification data and are placed based on automatic analysis of the building geometry. Having origin and destination, a Path Finding Algorithm (A* Search Algorithm [3,4]) calculates the shortest path between these points and draws it on a map to guide the user to his destination. As the user intersects another beacon region, the new user’s current location and the path are updated.

## 2. State of Art

There are diverse technologies that can be employed in Indoor Location Systems (ILS), an overview can be checked at [5,6]. Table 1 outlines the pros and cons of the main ones, adapted from [5]. The following categories are considered: Tracking Accuracy: considers the location precision that the device can return to the system; Maintenance Effort: describes cost and human effort in maintenance and updating the equipment; Industry Uptake: refers to how quickly the ILS is acquired by customers in the global market; Security and Privacy: represents Security and Privacy that this ILS can provide to the system; and Installation Cost. Comparing all the evaluated categories Beacon technology presents the best average between Pros and Cons and is chosen as the Indoor Location technology for this project.

A Beacon is a small Bluetooth Radio Transmitter that repeatedly broadcasts a synchronous Bluetooth Low Energy (BLE) signal in a restrict region/area. Each signal contains configurable data that can be received by a smart device. Beacons are the most recent indoor location technology, it is easy to configure and to maintain. It has its own Application Programming Interface (API) for the developers that want to implement an ILS. The security and Privacy of the users are safe, and the cost of each equipment is not expensive. Both beacon types, Estimote, and BlueCats, have very similar characteristics so we are sure that both would fulfil all the requirements of this project. Since the price and the characteristics of both beacons are similar, we chose the Estimote beacon because the design of the product holds a better look.

### 2.1. BIM Models

A Building Information Model (BIM) is a 3D detailed geometric description of a facility and of its contents: mechanical systems, furniture, and equipment. Information can be associated with the physical entities, but also to the spaces, e.g., their function or timetables. A parameterized BIM model is aware that a specific door is a double-leaf glass door, has level 1 fire-rating, connects corridor 1S to room 1S04, and that room is a 20-seat classroom. BIM is also a new process of work and collaboration for the Architecture, Engineering, and Construction (AEC) industry. BIM implementation is robust, in the first stages (design and construction) of the building life-cycle, and weaker in the final stages: operation and decommissioning [7]. In most new facilities, a BIM model is developed during the design phase and in some cases updated during construction. In the worst case, this model will hold a description of the geometry and materials, in the best-case relevant equipment, room names, and a description may be available.

Autodesk’s Revit [8] is one of the most widely used BIM softwares, and, as like most of them, it is built over a database. It is accessible with API [9]. NET compatible languages such as VB.NET and C#, renders it possible to connect the model to external information systems and import and export building-related information. The Dynamo [10] language gives easier access to Revit’s API through visual programming. In the case of relatively simple applications that don’t demand high performance, Dynamo allows for quicker development, although the maintenance cost is higher.

### 2.2. User Location/Orientation Systems and Mapping Applications Using Similar Technologies

Integration of BIM and sensors installed on buildings is one of the most relevant research topics in building construction and management, both for the industry [11] and for the academia. This dialogue meets with several difficulties, the major being the lack of open/common standards for sensor data exchange [12], and the current incapacity of BIM’s open standard IFC (Industry Foundation Classes) in handling and storing sensor data [13]. There’s ongoing research focuses on the development of IFC scheme to include sensor data [14], while discussing alternatives: Alves [15], also discusses sensor data-BIM integration in detail and proposes languages developed for the purpose: Building Information Modeling Sensor Language (BIMSL).

Works such as [16] develop a static connection between sensors and BIM model in a university laboratory, the authors of [17] integrate real-time sensor data used to assess the structural response of a bridge deck in its BIM model. Bottacioli [18] integrates data from several sources: environmental sensors (temperature, humidity, and electricity consumption), historical and real-time meteorological and BIM-based building energy modelling, to assess building expected and real thermal behavior. University campuses present excellent test-beds because of their centralized management, intensive utilization, and openness to research activities: the authors of [12] integrate sensors in a campus-wide, web-based system based on IFC and open messaging standards to gather energy, occupancy, and user feedback comfort while also providing other services to users such as room booking, location, and navigation assistance.

Regarding indoor mapping and navigation, Lin and Ho [19] describe a mobile app Indoor Location System using Beacon technology, applied to doctors and patients on a Hospital environment that shows patient position/location on a map, but does not employ BIM technology to feed the application.

On the specific topic of this paper, an indoor location associated with BIM [20] employ multimodal sensors for indoor location and use building geometry extracted from BIM model (restricted to one building level) to improve location precision. Shayeganfar [21] combines semantic and geometric information from BIM models and propose a navigation solution to be implemented on Android smartphones for people with special needs, but do not detail indoor location methods or implement the solution.

More specific research on the utilization of BIM for detailed navigation around obstacles (structural, openings, and furniture) with the goal of assisting in autonomous navigation [14,22,23] is being pursued, but only go so far as to demonstrate that BIM models contain the necessary information and that information can be extracted and used, but do not actually develop and test a complete navigation and indoor location system. The authors of [24] implement a hybrid BIM-Bluetooth System to locate workers and dangerous locations in construction sites.

This work’s novelty and contribution is not the development of a new framework, language, or platform for BIM + IoT (Internet of Things) integration, but a standalone mobile application which harvests geometric and semantic building information, including installed Beacon location and configuration from a BIM model.

## 3. Proposed Approach

The primary goal of this project is the development of an indoor guidance app that uses Bluetooth beacons for location and BIM for physical context information. The secondary goal is to obtain information about building utilization patterns, which is not covered in the present research work.

In the proposed app, once the user’s current location is obtained through the interception of a beacon region, the user has the option to insert the destination room. The mobile app then calculates the shortest path between the current location and the destination, using a Path Finding Algorithm. The calculated path is shown on the map and is updated step-by-step (when a new beacon is intersected) until the destination room is reached. If the user chooses not to follow the proposed path, the App will recalculate the way to the chosen destination.

**Figure 1:**
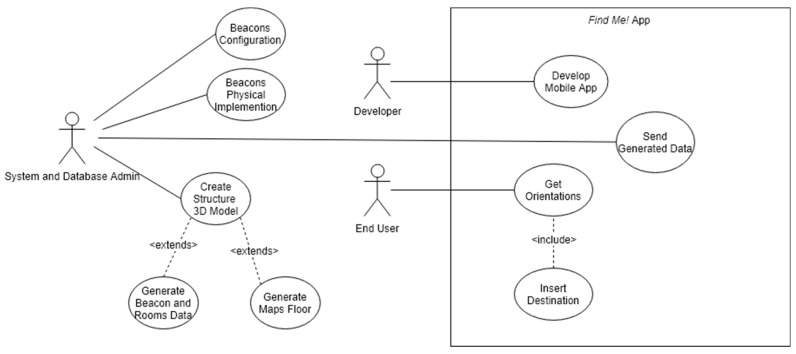
Find Me! uses a case diagram.

Each user role has a defined role as described in the list below and in Figure 1.

- **End User**: any facility user that installed the Find Me! App on an Android smartphone.
- **System and Database Administrator**: builds or adapts a 3D BIM the model of the building; generates maps and input data to the mobile App, e.g., facility geometry, room, and beacon location; configures and installs the beacons in the facility.
- **Developer**: develops and maintains the mobile App Find Me! code.

**Figure 2:**
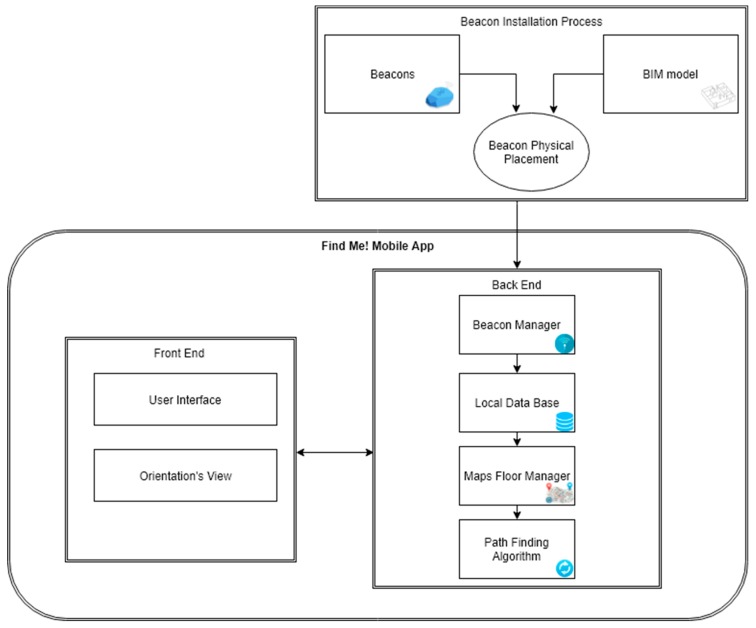
Find Me! architecture.

The Find Me! project has three main system modules, represented in Figure 2 and described below:

- **Beacon Installation Process**: The initial installation starts by finding the minimum number of beacons and their location considering the facility layout extracted from the BIM model. The beacons must be configured, placed in the facility and inserted in the BIM model.
- **Front-end Mobile App**: The user interface (UI) side where the user inserts the Destination, configures Options and receives dynamic orientation: the Map with the optimized path; Crucial Orientation Photos to confirm the user is in the right way.
- **Back-end Mobile App**: Contains a Local Database that stores beacon and room location, automatically extracted from the BIM model. The Beacon Manager scans for beacons and manages data from each intersected beacon signal. The Map Manager manages and configures the map(s) returned to the user interface. Beacon, room, stair, and elevator location and map images are generated by the BIM model and stored locally when the app is installed on the user’s smartphone. The Path Finding Algorithm calculates the shortest path between the user and the destination.

### 3.1. BIM Models

In the current work, ISCTE-IUL’s facility management office has been developing a BIM model which is being used to feed maps, room listings, and locations, as well as beacon locations. This proves a significant advantage because since the BIM models are based on a database that can be queried and updated (currently not in real time) with information to and from the app. To build a visual-based navigation system, easy to read drawings are needed for the UI, and information about rooms, paths, building entrances, stairs, and elevators are needed for the pathfinding engine. Getting this information in a format that can be consumed by the application is time-consuming. Building Information Modelling can significantly facilitate this task.

The BIM methodology depends on common, interoperable formats, but also on the controlled information sharing that breaks down information silos. In the current case, a BIM model of the campus is being developed by the campus Facilities Management team [25]. This model includes the complete geometrical description of the building including all room names. To make it useful to this application more information was added, namely: elevators and stairs were modeled, beacons were modeled with all relevant location and all areas of the building were parameterized as walkable and non-walkable. The software used for the development of the BIM model was Autodesk’s Revit version 2019.

Dynamo scripting language was used to extract lists of rooms with their coordinates, name, floor, and building; a list of beacons with coordinates and ID information and the coordinates of the nearest stair and elevator, and automatically generated images of the walkable regions in each floor.

### 3.2. Beacon Installation Process

Bluetooth Beacons are the most expensive component of the navigation system, so optimization of their number, location, and signal configuration are essential. Prior to installation, beacons must be configured both physically and in the BIM model. Estimote beacons can be setup to follow the Estimote or the IBeacon protocol [19]. This protocol was developed by Apple in 2014 and comprehends the following signal broadcast and beacon identification parameters:

- Signal Broadcast
  o UBroadcasting power can be set from −40 dBm (minimum) to +4 dBm (maximum) corresponding to a range between 2 and 70 m if no obstacles are present between Beacon and receiver.
  o Advertising frequency of the transmission packets, which can be configured from 100 ms to 2000 ms. A lower frequency and broadcast increase battery life. We use a parameterization of 1000 ms.
- Beacon Identification Parameterization:
  o UUID is a 16 byte identification, usually represented as a string such as “*B9407F30-F5F8-466E-AFF9-25556B57FE6D*”;
  o The major number is a 2 byte, or an “unsigned short”, i.e., a number from 1 to 65,535;
  o Minor number: 2 bytes, same as Major.

The protocol is suitable for this project because in each BLE packet three fields are sent thus providing the ability to have more combinations, create a beacon hierarchy and be more specific when identifying beacons’ signal origin and provide context information.

Every beacon must be pre-configured before deployment, by creating an account at the Estimote Cloud website, where the beacons are claimed and configured. Beacon Name follows a nomenclature was defined: ED + [Building number] + P + [floor number] + [Alphabet letter] to facilitate beacon identification. An example of a beacon name is ED1P1C: Building 1; Floor 1; third beacon. All beacons are configured with the same UUID to create a private beacons mesh, which prevents the addition of external beacon by other users. Major and minor fields differentiate beacons inside the mesh Parameterization is applied to beacons through the Estimote mobile app. A smartphone using internet access and Bluetooth connections work as a gateway between the Estimote Cloud and the beacons. 

### 3.3. BLS Signal Propagation Tests

In the current approach, it is admitted that in some locations the user may have to move to receive a signal from a beacon (e.g., in the middle of long corridors), but it is not admissible that a user receives a wrong location because the stronger intercepted signal does not correspond to his position, but, for example, from a lower floor. Signal propagation must be roughly understood, particularly interaction with building elements-walls and floors—and their materials:concrete; masonry; light plasterboard; or glass walls, as well as architectural features such as mezzanines. To this end, signal propagation and interference tests were performed in the pilot building. The tests conclusions tuned the Beacon Installation Process Heuristics detailed bellow. The main questions being investigated are:

- Is there BLE signal overlap from beacons on the same floor?
- Is there BLE signal overlap between floors?
- Is there BLE signal overlap in long corridors?
- Is there BLE signal overlap between floors in mezzanines?

To assist the tests, the *NearestBeacons* mobile application, based on the Estimote API [26], was developed. This auxiliary application scans and detects BLE signals and displays the following information about each intersected signal, on the smartphone screen:

- Major and Minor integer-identify of the beacon;
- RSSI—Signal strength value that depends on broadcasting power, distance, and obstacles [27];
- Measured Power-expected RSSI at a distance of one meter to the beacon [28].

The app ranks the intercepted signals according to signal strength (RSSI).

**Figure 3:**
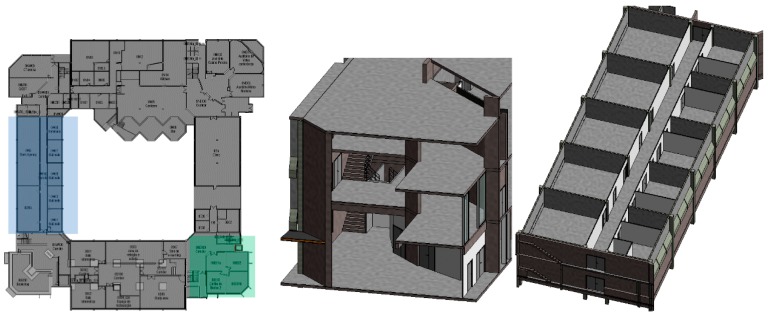
Location of the two signal propagation zones in building floor plan in ISCTE-IUL’s Building 1 (**Left**), the perspective of the mezzanine zone, marked in green (**Middle**) and of the corridor zone, marked in blue (**Right**).

**Figure 4:**
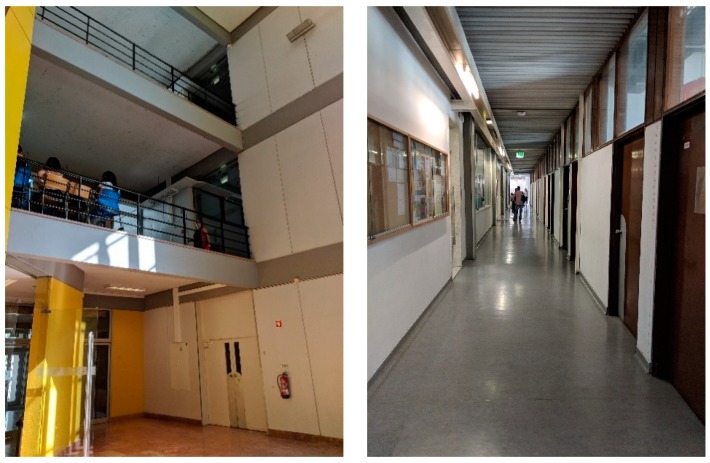
Signal propagation test locations: mezzanine (**Left**) and corridor (**Right**).

In the first test, all beacons were configured with a broadcast power of −16 dBm which should guarantee 7 to 10 m range in the unobstructed ground. The pilot building has glass, plasterboard, masonry, and concrete walls, stairs, mezzanines, and solid concrete slabs. Several propagation configuration situations were studied. Figure 3 displays the test areas: in blue a 35-m long corridor and in green a mezzanine that spans three floors. In Figure 4 which shows the corridor and mezzanine. To prevent theft and facilitate installation and removal, beacons were placed near the ceiling, both were secured in cable trays or glued to the ceiling.

**Figure 5:**
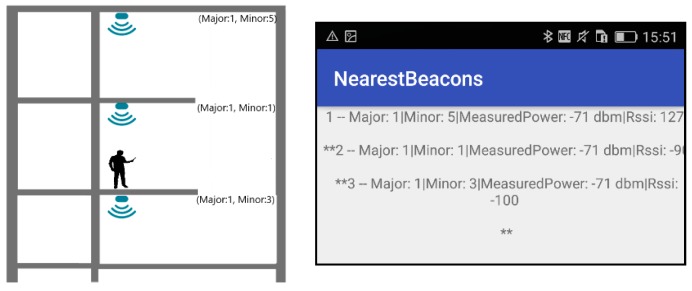
Test configuration in mezzanine (a green region in Figure 3) and a screen print of the corresponding NearestBeacons App.

**Figure 6:**
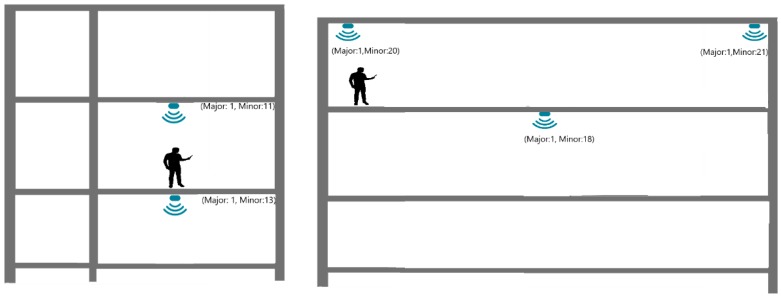
Test in the corridor (a green region in Figure 3): interference between floors with axis placed on the same vertical alignment (**Left**); interference between beacons placed along the axis of the corridor (**Right**).

**Figure 7:**
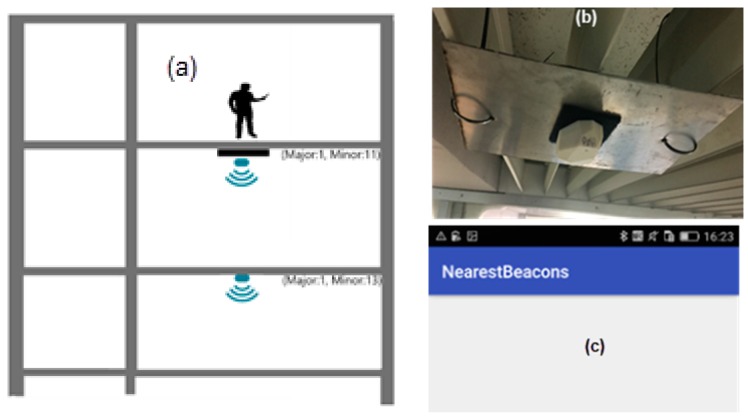
Test of signal blocking with a 20 mm × 200 mm × 2 mm steel plate between the beacon and the ceiling in a corridor (a green region in Figure 3): beacon installation (**b**) and NearestBeacons App results showing no received signal (**c**).

Presented test layouts and results are condensed, refer to [29] for a complete description. Figure 5, Figure 6 and Figure 7 displays three test configurations investigating signal superposition between floors (upwards and downwards), propagation in open areas, both along corridors and in mezzanine balconies. Also tested is the installation of a 200 × 200 × 2 mm steel plate between the beacon and the ceiling to redirect the signal downwards.

The propagation tests results led to the following conclusions:

- In the three-floor mezzanine, the signal power attenuation is much smaller and in some test configurations and user positions (not shown in Figure 5) the signal from the lower floor is stronger than the signal from the tester’s floor leading to an incorrect user position.
- Tests with beacons vertically aligned on floors separated by 0.3 m thick reinforced concrete floors (Figure 5) showed that these do not block the signal entirely, but attenuate it significantly leaving no doubt on the correct location. Variations of tester and beacons lead to similar results.
- In tests along the axis of a corridor (Figure 6) only the nearest-same floor as a tester-beacon was received.
- Tests with a 200 mm × 200 mm × 5 mm steel plate (Figure 7) installed between the beacon and the ceiling showed that it successfully blocks the BLE signal from propagating upwards.

With these simple tests, the mechanics of BLE signal propagation on the pilot building was gathered. Other tests on the same building could provide further insights into the influence of light walls with steel structure, false ceilings or cable trays, but these were not determinant on our pilot. The following list contains the major conclusions of the tests:

- In areas with vertical communication such as stairs and mezzanines signal strength must be decreased to obtain a smaller range;
- In vertical alignments separated by a concrete floor, a beacon per floor is necessary,
- In long (15–20 m) corridors there is no risk of signal interference, and a beacon may be placed at each end of the corridor;

It should be noted that in buildings with other structures and construction systems different tests might be necessary.

Beacon Placement Rules define an equilibrium between the location system’s cost and usability. If many beacons are installed, each with low power, user position will be very accurate with high installation and maintenance costs. Main optimization factors are building size and number of rooms, space distribution, exterior accesses and wall, and floor materials. Automated analysis of the building geometry over its BIM models identifies:

- entry/exit points;
- circulation areas such as corridors and halls;
- intersections;
- stairs and elevators; and
- the center of long corridors.

**Figure 8:**
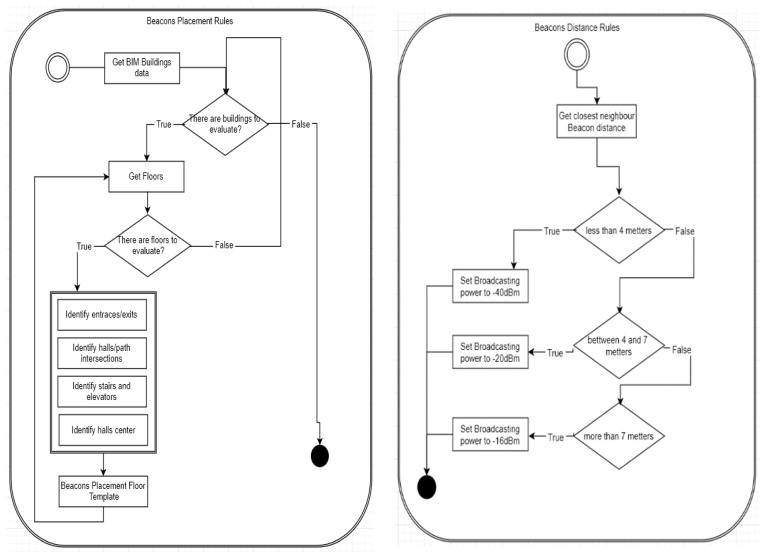
Beacon placement rules diagram (**Left**) and beacon signal power rules diagram (**Right**).

A systematic approach was applied to extract information regarding entry/exits points from BIM models. At points where user presence is to be collected, a beacon is placed. So building geometry information extracted from the BIM model is used to set a list of locations for the beacon placement as illustrated in Figure 8. Basically it extracts entry/exit points and places where decisions have to be made and calculates distances for beacons power output configuration, following rules in Figure 8. After locations are determined, they are inserted in the BIM model. Then, distances between beacons are extracted from the BIM model, and the beacon signal strength is configured. BLE signal overlap between beacons is possible in some situations, but the FindMe! App uses the NearestBeacons App sort function to rank highest to lowest RSSI values, in cases where more than one signal is intercepted. This, conjugated with the presented heuristic assures to a high degree that the correct location is indicated.

The oldest building of the ISCTE-IUL campus was chosen as a test-bed. Building Sedas Nunes has three above-ground floors, four main entrances/exits, four stairs serving all three floors and around 300 rooms. It has the shape of an 80 m long square with a 20 m long interior courtyard. Rooms are named following the nomenclature of [floor number] + [cardinal point orientation of the wing] + [incremental number of the room]. For example room, 2E10 is located on the second floor on the East aisle of the building and sequentially is the fifth on the left side of the corridor.

Given the layout of the pilot building, with similar plans in all floors, circular circulation and vertical communication in the corners, application of these rules led to the following configuration: eight beacons for each floor, in the corners and middle of the corridors, a total of 24 beacons. Based on performed tests App always catch beacon signal and since we use a metal plate to cut propagation to upper flow there is no wrong information. We arrived at 100% accuracy process and based on the received strength signal using beacon location information the location precision has always below 0.5 m.

### 3.4. Building Information Extraction

Due to increasing BIM penetration in the construction industry, most new building designs are designed using the BIM methodology. Due to low building owners’ awareness of the potential of BIM in building management, this model is seldom transferred to the owner. However, Facility Managers are growing aware of the relevance and develop the model from existing 2D blueprints, which is the case of ISCTE-IUL which has transferred its blueprints to digital, 3D BIM format. This model is linked to the IT systems for room occupation and characteristics update via a unique room identifier key. All building-related data that provides the FindMe! App with physical context is extracted from the pilot building BIM file, modeled in the Autodesk Revit format. The model was upgraded to Revit version 2019, and a few adaptations were made to the geometry, namely correction and reparameterization of stairs and elevators and insertion of missing doors. Classroom and office information (occupants, department, and school) was imported into the model from the university’s academic management system using dedicated Dynamo scripts, and parameterization of all remaining areas, such as circulation hall, elevator, stair, and technical non-access areas, was manually added to the model. A Dynamo routine that scans each door in the model retrieves the identification of the room the door serves (i.e., opens to) and writes that id in the door “Mark” parameter was developed and applied to the model. This way navigation is facilitated since when a user wishes to navigate to a room he is led not to the center of the room but to one of its doors. Finally, a Revit family with identification and broadcast parameters was created for the beacons, which were individually inserted into the model, along with their alphanumeric information. These modeling tasks were performed by a trained BIM modeler in about two days, not counting with Dynamo routines development.

The next step is then to integrate beacons, doors, stairs, elevators locations, and their interrelationships into the FindMe app, and create two kinds of floor plans: one for user visualization and another for internal app navigation. This analysis and export are performed by a set of Dynamo routines that analyze the model and generate two Comma-Separated Values (CSV) files for the building and two 2D Portable Network Graphics (PNG) files per floor.

The Main Dynamo script for beacon and door/room tables production can be downloaded from [29,30], and displays two examples of 1000 × 1000 pixel 2D PNG files. All information is generated directly from the BIM model, including room identification. In the navigation plan, the red area indicates zones where navigation is allowed. The allowed areas correspond to “rooms” in the BIM model that are tagged as walkable, depending on their classification, as previously. A first Dynamo script extracts stair and elevator location (coordinates and building floor) and the floors each of them serves. Then two other scripts, shown in [29,30] extract beacon and door location and internal parameters and determines the stair and elevator closer to each beacon and door and generate the CSV files. All coordinates are converted from meters to a 1000 × 1000 pixel grid. In the pilot building, this corresponds to approximately 10 pixel/meter, which is a good compromise between location precision/image quality and file size. The origin of the grid is coordinated with the PNG files view of the floor plans.

**Figure 9:**
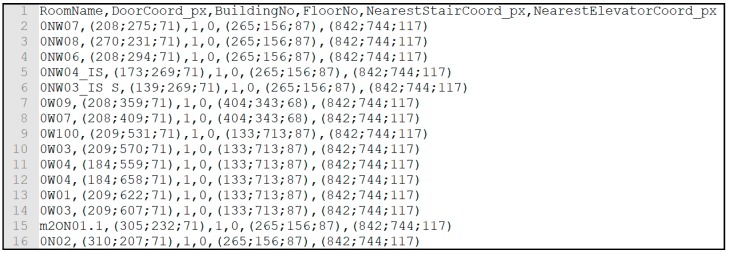
CSV file containing room information.

**Figure 10:**
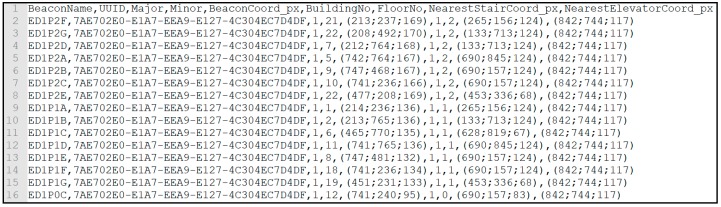
CSV file is containing beacon information.

Figure 9 and Figure 10 display CSV files containing room and beacon information. In both files, each entity is identified, in the case of rooms through its number, in the case of beacons through: Name; unique UUID; Major integer (which identifies the building number, in this case always 1); Minor integer (which identifies the beacon inside the building, starting at 1). Then, the room/beacon location coordinates; building number; floor number; closest stairs coordinates; and closest elevator coordinates are provided.

## 4. Find Me! Mobile App

The application comprises several modules which are summarized in Figure 2 and described below.

The Beacon Manager handles a beacon signal that is intersected while the user is moving in the building. Estimote’s Software Development Kit (SDK) includes an API to handle the BLE connection to all intersected beacon signals and processes the data packets. This SDK is added as a dependency of the Android Studio project and version 1.4. was used. After adding the dependency, the Android Studio compiler automatically synchronizes the gradle, and the Estimote API is ready to use in the code. To start beacon monitoring, the *connect()* method is called to switch on the smartphone BLE receiver. Besides that, in this method it’s possible to filter which kind of beacons to intersect, creating a private Beacon Region instance.

The Beacon Configuration checks the intersected beacon’s UUID value with the app-recorded one: 7AE702E0-E1A7-EEA9-E127-4C304EC7D4DF. It’s intended to get all beacons in this mesh, so the third and fourth arguments are set to null to intersect beacons with different major and minor values.

After calling the *connect()* method, the app is able to receive BLE packets by calling the *BeaconMonotoringListener()* method. This method can be triggered in two cases: when entering and leaving a beacon region. The *onEnteredRegion* method is triggered when more than one BLE packet is received. After that, it’s assumed that the user is in a beacon region if BLE packets are received. This method also gives access to an intersected beacons list (ordered by the closest beacon to the farthest, the RSSI value). For each beacon, the UUID, Major and Minor values are received. In the current implementation, these values are sent to the app database as a query, to identify the coordinates of the beacon and the user. If the receptor, the smartphone, stops receiving that kind of packets it will trigger the *onExitedRegion*, and it’s assumed that the Beacon Region is abandoned. The Beacon Manager, when initialized, runs as a background thread that always looks for BLE packets from the moment the user runs the App until he/she closes it. This feature allows the update of the path during utilization.

A Local Database was implemented to allow the app to run without internet access which could compromise the main objective of this project. A full local database was chosen over an external database. Floor maps, rooms, and beacon data from the BIM Model, as shown in Figure 9 and Figure 10, are stored locally.

The Maps Manager entity is responsible for managing all the map floors (PNG files) that need to be returned to the Path Finding Algorithm, according to the current location and destination of the user. The Map Manager extracts, from the returned query (*getCurrentLocation* and *getDestination*) in the Local Database, the building and the floor number of each Location. After that, a string is created with the extracted values, and the final format is going to be: “edificio” + [building number] + “piso” + [floor number]-example: edificio1piso1. Having created both strings (one for origin and the other for destination), this entity validates if both strings are equal. The Map Manager follows a rule to make sure that the returned map floor is the correct one. This rule is based on two critical aspects: both strings hold the same value: it means that the user is in the same building and floor than the destination, so the Map Manager just needs to return one floor map. If string values are different: the destination is on a different floor of the current Location of the user. Considering that every floor is connected by stairs or elevators, this entity returns two map floors.

**Figure 11:**
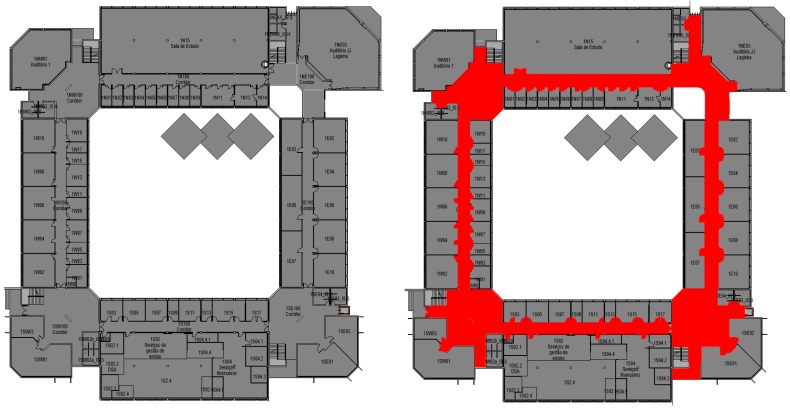
Floor plans generated from the Building Information Model (BIM) model for user visualization (**Left**); internal app pathfinding algorithm support with walkable areas in red (**Right**).

The Path Finding entity goal is to calculate the shortest path between two locations. This algorithm receives, as input, a map floor, current location and destination of the user. BIM model provides information on entry/exit points and internal path intersections and a graph with dependencies is created. In spite of relative building complexity for human users, the numbers of nodes of this graph network are much smaller than a city street map. In the university pilot case, graph nodes where around 100 for the complete campus and the algorithm applied in the mobile device provides a response in few milliseconds. After evaluating each combination of pixel colour of the map floor, which is converted into a pixel matrix, it returns the optimized path, a pixel array. The first step of this algorithm is to map which areas/paths are walkable (in order to avoid forbidden areas, walls, voids, etc.) and then calculate the shortest path between the two locations. The walkable map floor, which is an input of the app from the BIM model, has some pixels marked in red (Figure 11) which are converted into a matrix of pixels. Each red pixel matrix is set as walkable. The A* star algorithm [31], adapted in [32] tries to find all possible options to reach the destination taking into account the coordinates of the current location and of the destination into the pixels matrix, validates all the paths from the start pixel to the final pixel, thought the walkable paths already set on the first step. After this, it picks up the path that holds the lowest cost [32], which means that is also the shortest path between current location and destination. In the end, the lowest cost path is returned to Map View entity to be painted above the map floor to show the user the path that he/she needs to run on that floor to reach the destination. In case the Path Finding Algorithm returns no pixels array, it is not possible to reach the destination.

Users’ movement is checked at beacons position range (with an average error of less than 0.5 m) based on beacon position and received signal power. Between two beacon signal interception, information from mobile device accelerometer (speed and direction) and gyroscope (mobile device orientation) are used, as described in previous authors’ work on mobile devices [33]. Based on the internal sensors orientation and movements provide a real-time user position using algorithms developed [34] for Android mobile devices. In our case, taking into account average beacons distance (30 m), the tested cases show average position precision below 1 m with a maximum value of 2 m. These measurements were performed in a control test environment measuring App location against real user location. Movement detection between beacon signal depends on mobile device sensors and distance because of dead reckoning known limitations [34].

## 5. Mobile App Front End

The app’s Front End comprises several views, described below.

**Figure 12:**
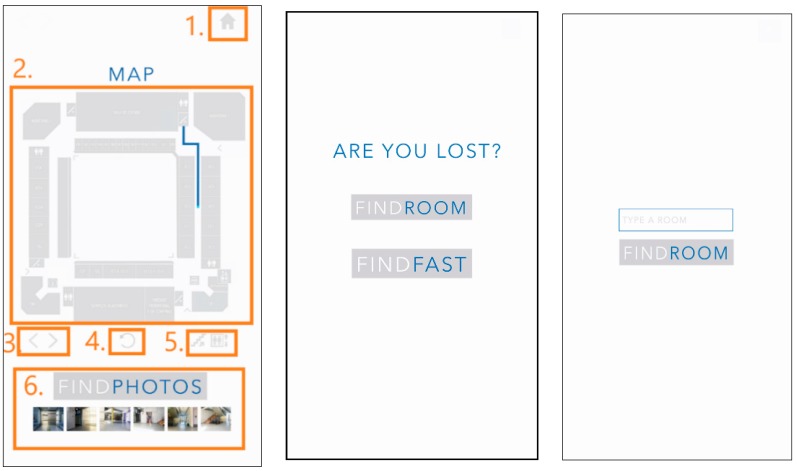
Floor Map view screen description (**Left**), Choose Destination options (**Center**) and FINDROOM option (**Right**).

In the Orientation View all the orientations are provided to the user. These orientations are dynamic, so every time the user’s smartphone intersects a beacon, it is updated with new orientation. The screen shown to the user can be observed in Figure 12. The option in the figure are described below 0.

Home (1): This button goes back to the start screen, where it is possible to change the inserted/chosen destination.

**Figure 13:**
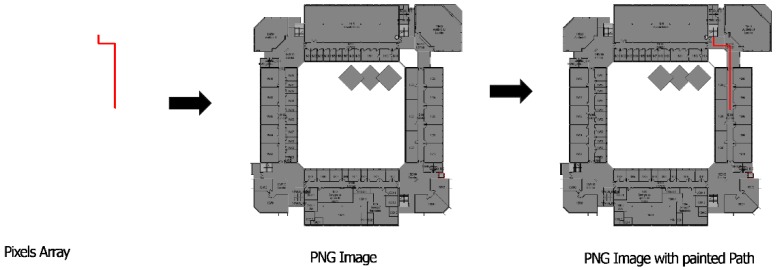
Floor Image View set process.

Map View (2): It’s an Image View element that shows the user the path to reach the destination. This entity receives a PNG image from Map Manager and an array of pixels from Path Finding Algorithm that corresponds to the calculated shortest path. The PNG image it’s converted to a BitMap and a method from Orientation’s View code is called to match the array of pixels and paints them over the map, creating a new PNG image that is set on the Image View element, as seen in Figure 13.

Change Floor Arrows (3): this button group will only appear when the user’s floor is different from that of the destination: selecting the right arrow button requests Map Manager to show the map floor image that corresponds to the destination floor. When left arrow is selected, it goes back to preview the current Location map.

Rotate (4): Rotates the Map View (2) element 90° degrees to help the user get a better perspective of the map.

Elevator and Stairs (5): this button group will only appear when the user’s floor is different from that of the destination: this feature enables the user to choose between using the elevator or the stairs from the current location floor to the destination floor. The default is the stairs, changing to elevator makes a request to Path Finding Algorithm to calculate the path using the nearest elevator.

Find Photos (6): This horizontal image scroll view displays photos of the path in a way that the user can confirm he/she is on the right path to the destination. There is a group of photos associated to the locations the user needs to go through. To populate the Find Photos element, the *getImagesScroll* method is called to check if there are photos intersecting the path array of pixels. This method receives *LinearLayout* (to render this element), a string format which confirms in each building, and floor, the array of pixels, an Integer list to manage the id’s of each image, the activity where it is going to be rendered and the listener that will be aggregated to each image.

The User Interface entity is responsible for asking the user for the room that he/she wants to reach into the building—the destination. The user picks one of two options to choose a destination: selecting a “Fast Room” or manually write a destination. The FINDFAST option offers the ability to choose common destinations such as Water Closet (WC), restaurants, Automated Teller Machine (ATM), academic services and security station spots. These destinations can be parameterized in the BIM model.

**Figure 14:**
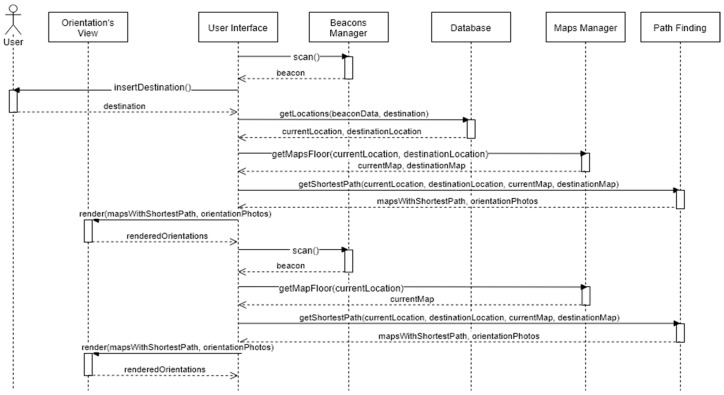
Sequence Diagram of Orientations Process.

Figure 14 shows how the different entities communicate with each other to give the right orientations to the user, in a sequence diagram. Note that the BIM model component isn’t implicit in this diagram, because this entity is only needed when there are changes in the building data or beacons.

When the Find Me! App runs, it automatically scans for a beacon. If a beacon is intersected, Beacon Manager Entity returns the intersected beacon data (UUID, Major, and Minor values)—that will be converted after as the current location of the user and triggers the next activity/screen of the mobile App: Insert Destination. The user enters the intended room and a query runs on the database returning the current and destination locations. The Map Manager is called in order to provide the floor maps of each location or just one map if origin and destination are on the same floor. The A* algorithm runs and returns the shortest path, and the Orientation View renders it on the map. While the user is moving, the App keeps scanning for other beacons with the goal of getting an updated current location.

## 6. Evaluation

Every year, ISCTE-IUL receives an average of 1150 new students distributed in sixteen graduate courses and approximately 200 Postgraduate, Master, and Ph.D. degrees. A high percentage of these students do not know how the facilities are organized as a physical structure and when the moment to go to a specific room comes, they need to ask how to get there. After meetings with the ISCTE-IUL Technology and Information department, it was decided and confirmed that ISCTE-IUL is going to be used as a validation approach to this concept.

Building Sedas Nunes, used to test Beacon Placement and described in that section, was also used as a pilot for the navigation. Individual testing was performed from June to August 2018, where new students were asked to use and evaluate the App. We asked these students to find a room with and without the usage of the App. On average, the 50 tests cases show a 30%reduction of time to arrive at the room. Real tests are planned for the start of the next year, where around two thousand new students will arrive on campus and need guidance towards administration, classrooms, etc. At the campus, QR codes will alert the students for App download, and the App will also be used to send context information to the new students.

It was decided to split the tests into three scenarios: in the first, the origin and destination are on the same floor, in the second one, they are on different floors, in the third the origin is in a place not covered by a beacon region.

**Figure 15:**
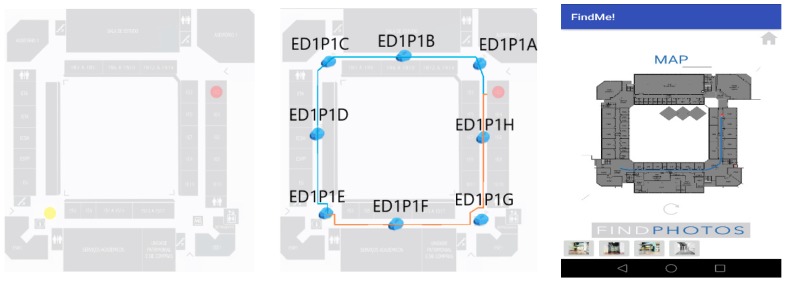
Test 1—yellow point marks the start, the red point marks the destination (**Left**); path taking into account two path options with beacon signal information (**Center**); an example of a screen App with map path and the possibility of displaying photos (**Right**).

In Test 1 the current location floor is the same as the destination floor, so there is no need to use stairs or elevator and the App is expected to render one map floor. The user starts on the Southwest corner and chooses room 1E02, on the East wing, as shown in Figure 15. This case was tested with 18 subjects and results showed 100% guidance and users did not report errors. Beacons were always intercepted by mobile devices and correct guidance performed.

**Figure 16:**
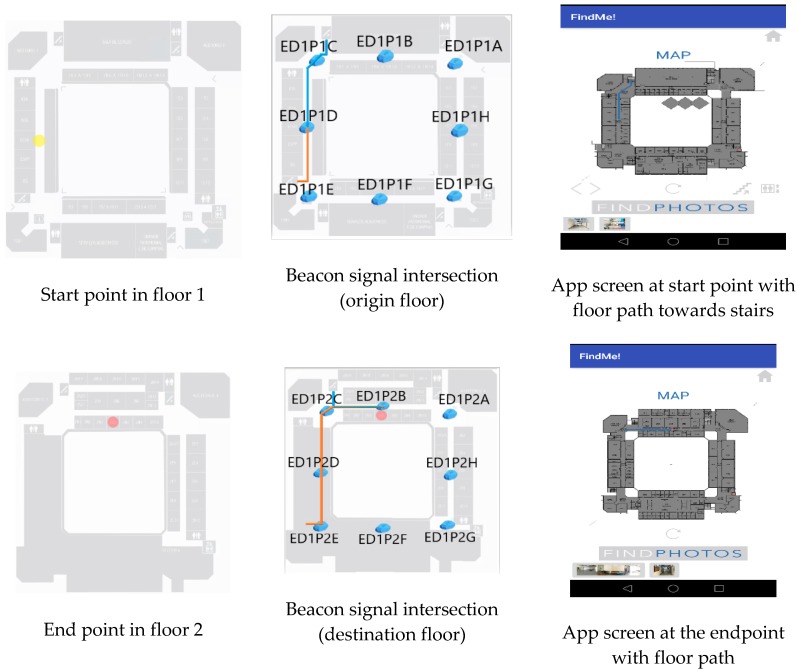
Test 2—Origin and destination (**Left**), paths using the stairs with beacons intersection signal (**Center**) and App screens (**Right**).

Test 2 was performed to evaluate the performance of Find Me! App when there is the need to change floors. This can be done by stairs or elevator. The test was split into two subtests, one for each option. In both subtests, the start location is West wing side, first, and the destination is room 2N05, in the North wind, second floor. Several path configurations can be performed using an elevator or stairs. Both tests run successfully and the test using the stairs is shown in Figure 16. We had 23 subjects in our testing with 100% beacon correct location acquisition and correct App guidance in all cases to the right room, with associated images.

**Figure 17:**
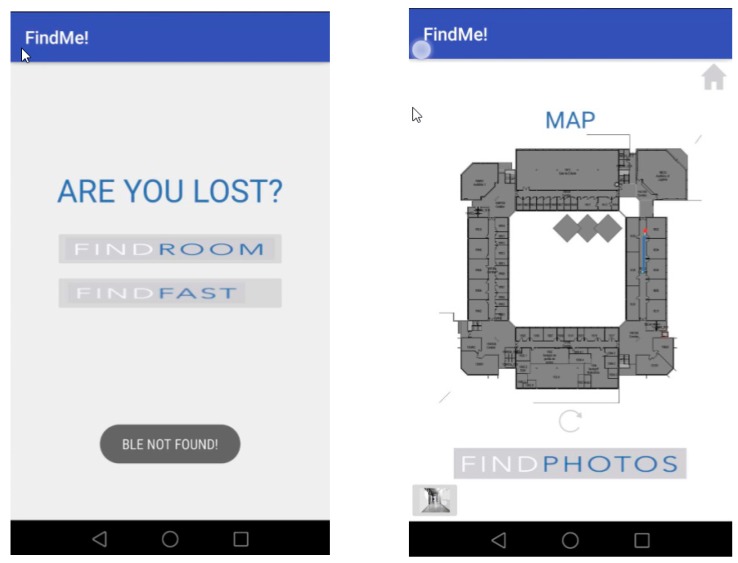
Test 3—at the starting point with no beacon signal (**Left**) and when a beacon is intersected, location is acquired, and guidance process starts.

Test 3 was planned to evaluate the Find Me! App behavior when the start point is in a building area not covered by a beacon. The origin floor was floor 1, and the destination room was 1E02. The users ran the App, and the Find Me! stayed locked in the Insertion Mode options screen, showing the message: “BLE NOT FOUND”, as shown Figure 17. The user started walking north and after a few meters he intersected the beacon ED1P1H. Having received the BLE signal, the Insertion Mode options screen unlocked the available options, the user typed the room destination and navigation started. Having a beacon in each intersection and middle of the corridors worked well in this case. We had nine subjects in our testing with 100% beacon correct location acquisition and App guide all cases to the right room, with associated images.

In the app demo and promotion video available at https://vimeo.com/303014540 or https://goo.gl/VQt1wG. It is possible to verify the app performance with real-time guidance and environment images and the real-time path update/recalculation as user reaches new beacons in the corridor halls.

## 7. Conclusions

An implementation of a Bluetooth Beacon indoor location and pathfinding solution that can be customized and applied to any type of building, with the condition that a 3D BIM model of the building is available, is described in this work. The solution is developed as an Android mobile app which can be delivered in a smartphone or a tablet/kiosk.

The application was tested in a university campus building with a circular geometry which is frequently challenging to newcomers.

The major innovations on this work are the process of Bluetooth Beacon placement planning using the geometry of the building which is described by a BIM model, the combination of beacon location information with available buildings maps and information, reducing the tedious task of gathering room information and location. Another contribution of this research work is that user movement can be registered passively (without user identification). This critical information can be analysed to extract knowledge about users’ movements inside public buildings. Also, this approach allows a simplified implementation process for indoor location and guidance, where location precision is based on beacon configured propagation radius and location uncertainty (zones without beacons coverage) is handled by building geometry using information of entry and exit points. Between two beacon signal zones, the solution assumes a regular walk based on pre-defined velocity or based on previous user walking velocity.

This approach can be easily replicated in other buildings with the introduction of new maps and calibration of beacons signal range to account to different propagation conditions. Also, there is the possibility of association of information services through the beacons, e.g., beacons near the library may advertise events, canteen menus, available administrative services, or evacuation guidance for emergency situations. Building changes and constraints (areas under maintenance, room changes, closed exits, etc.) can be quickly generated from the BIM model and sent as an update to the app.

## 8. Future Work

One recurring difficulty in the utilisation of the app is when the user is in a location outside the range of a beacon. One of two options can improve this situation: incrementing the number of beacons (which will increase the project budget) or returning, in mobile App, information messages for the user to move along until he intersects a beacon region. On the next release, we expect to analyse the placement of Near-Field Communication (NFC) tags in room doors, where the user can pass the smartphone to receive their content and send it to the Find Me! App.

Another future development is the generation of reports that are working anonymously, gather information such as most searched rooms, building areas where users get lost, stairs/elevator preference, least searched rooms. This information can then be used not only to manage the building better but also to influence users into more sustainable and healthy options such as using stairs over elevators for short paths.

The application can also be used outside the building to preview a path without the need to be physically present. In this case, the initial room must be inserted, or the location must be selected on a map or 3D model. The flux will be like the described with the exception that the beacons will not update the path.

BIM models can also be used for beacon signal propagation if the wall and floor material properties are characterized, thus reducing or eliminating the need for signal propagation testing.

Finally, in this study, only one building was tested. If several buildings are included in the application, they can be modelled in one common BIM model or an interface between them must be designed in the data structure.

## Figures and Tables

**Table 1:** Pros and Cons of different Indoor Location System (ILS) devices.

	QRCode	NFC	GPS	WiFi	Beacon	Lidar	Ultrasound Systems
Tracking Accuracy	High	High	Low	Low	High	High	High
Maintenance Effort	Easy	Difficult	Difficult	Easy	Easy	Medium	Medium
Industry Uptake	Medium	Medium	Low	High	High	Medium	Medium
Security and Privacy	Medium	High	High	Medium	High	High	Medium
Installation Costs	Low	Low	High	High	Medium	High	Medium
